# Fourth-generation chimeric antigen receptor T-cell therapy is tolerable and efficacious in treatment-resistant rheumatoid arthritis

**DOI:** 10.1038/s41422-024-01068-2

**Published:** 2025-01-09

**Authors:** Yujing Li, Sujun Li, Xiaojuan Zhao, Jun Sheng, Lei Xue, Georg Schett, Ce Shi, Biliang Hu, Xingbing Wang, Zhu Chen

**Affiliations:** 1 Department of Rheumatology and Immunology, the First Affiliated Hospital of USTC, Division of Life Sciences and Medicine, University of Science and Technology of China, Hefei, Anhui China; 2 Department of Hematology, the First Affiliated Hospital of USTC, Division of Life Sciences and Medicine, University of Science and Technology of China, Hefei, Anhui China; 3 Department of Rheumatology and Immunology, the First Affiliated Hospital of Wannan Medical College, Wuhu, Anhui China; 4 Department of Internal Medicine 3, Rheumatology and Immunology, Friedrich Alexander University Erlangen-Nuremberg and Universitätsklinikum Erlangen, Erlangen, Germany; 5 Siweikang Therapeutics, Changsha, Hunan China; 6 Department of Hematology, Centre for Leading Medicine and Advanced Technologies of IHM, the First Affiliated Hospital of USTC, Division of Life Sciences and Medicine, University of Science and Technology of China, Hefei, Anhui China

**Keywords:** Autoimmunity, Biological techniques

Dear Editor,

Rheumatoid arthritis (RA) is an autoimmune inflammatory disease characterized by symmetric synovial inflammation leading to progressive disability.^1^ Treatment of RA has been revolutionized by the development of monoclonal antibodies against cytokines (e.g., tumor necrosis factor-α (TNFα) and interleukin-6 (IL-6)) as well as B-cells (e.g., CD20-targeted antibody rituximab), resulting in better disease control.^2^ However, up to 30% of RA patients still escape therapeutic responses despite several cycles of immunomodulatory drugs.^3^ Such patients are referred to as “difficult-to-treat (D2T)” RA according to European Alliance of Associations for Rheumatology (EULAR) definition.^3^ Here, we report on the efficacy and safety of a new, autologous, fourth-generation CD19-targeted chimeric antigen receptor (CAR) T-cells that secrete antibodies against IL-6 and TNFα (CD19/aIL-6/aTNFα) in treating D2T RA (Fig. 1a, b).

**Fig. 1:**
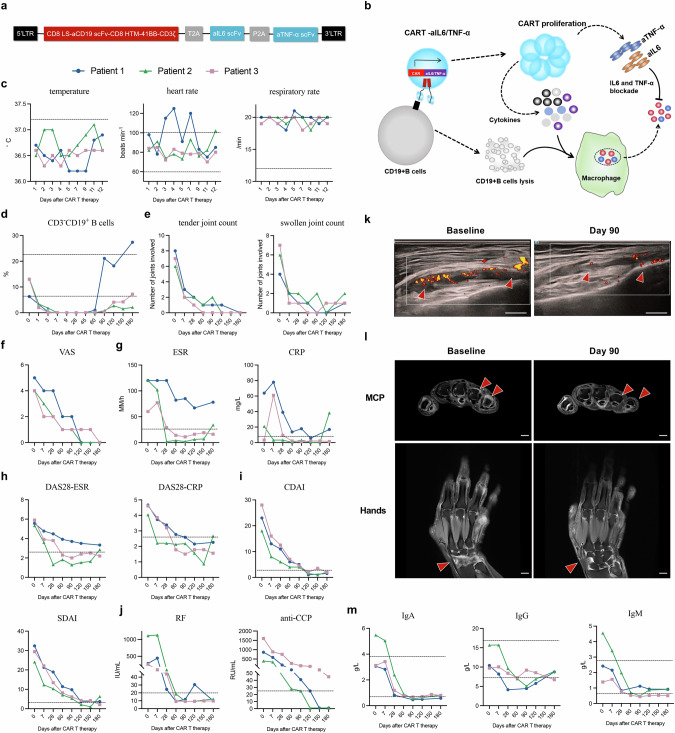
Clinical safety and efficacy of CD19/aIL-6/aTNFα CAR T-cells in RA. **a** Design of chimeric antigen receptor (CAR) construct and sequences of anti-IL-6 single chain variable fragments (scFv) and anti-TNFα scFv. **b** Schematic illustration of CAR T-cells depleting CD19 B cells and secreting scFv of immunoglobulins against IL-6 and TNFα (CD19/aIL-6/aTNFα CAR T-cells). **c** Body temperature, heart rate and respiratory rate taken at the same time of the day after treatment with CAR T-cells. **d** Numbers of circulating CD19^+^ B cells in the patients’ peripheral blood. **e** Effects of CAR T-cells on tender and swollen joint counts (TJC, SJC). **f** Physician global assessment of disease activity using visual analog scale (VAS). **g** Effects of erythrocyte sedimentation rates (ESR) and C-reactive protein (CRP) levels. Disease activity scores-28 (DAS28) based on ESR and CRP (**h**) and clinical disease activity index (CDAI) and simplified disease activity index (SDAI) (**i**). **j** Serum levels of rheumatoid factor (RF) and antibodies against cyclic citrullinated peptide (CCP) at baseline and during 6-month follow-up. **k** Representative images of ultrasound Power Doppler (PD) signal (arrowheads) in the knee joint before and 3 months after CAR T-cell therapy (patient 2). Scale bars, 1 cm. **l** Representative MRI scans showing improved synovitis (arrowheads) of the hands and metacarpophalangeal joint (MCP) at baseline and 3 months after CAR T-cell treatment (patient 2). Scale bars, 1 cm. **m** Effects of CAR T-cell therapy on serum levels of immunoglobulins G (IgG), A (IgA), and M (IgM). Dotted lines, cut-off values.

Three patients with RA, refractory to multiple conventional and biological agents, were treated with these new fourth-generation CAR T-cells. The study was approved by the ethics committee of the First Affiliated Hospital of University of Science and Technology of China (2023KY-379). All the patients provided written informed consent in accordance with the principles of the Declaration of Helsinki. The ability of CD19/aIL-6/aTNFα CAR to release antibodies against IL-6 and TNFα was tested in vitro (Supplementary information, Fig. S1a–d). Detailed demographic and disease-specific characteristics of the patients are shown in Supplementary information, Table S1. Briefly, patient 1 is a 49-year-old, patient 2 a 52-year-old, and patient 3 a 56-year-old woman, who had active, severe RA with disease activity score 28 (DAS28)-erythrocyte sedimentation rate (ESR) scores at 5.57, 5.34, and 5.90 units, respectively. All patients had previously been exposed to glucocorticoids and multiple disease-modifying antirheumatic drugs (DMARDs), including methotrexate (3/3), hydroxychloroquine (2/3), leflunomide (2/3), iguratimod (2/3), etanercept (2/3), adalimumab (1/3), tofacitinib (1/3), baricitinib (2/3), abatacept (1/3), and recombinant Human TNFα Receptor II: immunoglobulin (Ig) Fc Fusion Protein (1/3). After CAR T-cell treatment, all three patients discontinued biological DMARDs (bDMARDs). Baseline glucocorticoid dose was tapered to half (patients 1 and 2) and discontinued in patient 3. Hydroxychloroquine was maintained in patient 1 and patient 3. Non-steroidal anti-inflammatory drugs (NSAIDs) were taken on demand in patient 1.

In all three patients, CAR T-cell infusion was well tolerated and no serious adverse events occurred (Supplementary information, Table S2). Throughout the infusion, body temperature, heart rate and respiratory rate were monitored continuously and remained within normal range, with a short phase of mild tachycardia in patients 1 and 2 (Fig. 1c). No cytokine release syndrome (CRS) or immune effector cell-associated neurotoxicity syndrome (ICANS) occurred, as no fever, hypotension, headache, difficulty breathing, or neurologic symptoms (such as confusion, seizures, changes in consciousness or behavior) were observed in the three patients. After infusion, CD19/aIL-6/aTNFα CAR T-cells rapidly expanded in vivo, with CAR copies peaking on day 9 in patient 1, on day 21 in patient 2 and on day 14 in patient 3 (Supplementary information, Fig. S2a). CD19^+^ B cells vanished from the patients’ peripheral blood after 3 days in patient 1 and 7 days in patient 2 and 3 (Fig. 1d). Blood counts decreased in conjunction with conditioning treatment and quickly recovered during the observation (Supplementary information, Fig. S2b). Of note, patient 2 had late neutropenia after 28 days and 90 days, which has been described in conjunction with CD19 CARs.^4^ Granulocyte colony-stimulating factor was offered and the neutrophils went up afterwards. On Day 15 after CAR T-cells infusion, patient 2 developed mild COVID-19 infection and recovered after treatment with standard dose of paxlovid.

After CD19/aIL-6/aTNFα CAR T-cell treatment, the patients rapidly improved. The number of tender joints decreased from 8 to 0 in patient 1, from 6 to 0 in patient 2 and from 7 to 0 in patient 3 (Fig. 1e). The number of swollen joints decreased from 4 to 1 in patients 1, from 6 to 2 in patient 2 and from 7 to 1 in patient 3 (Fig. 1e). Physician-based assessment of global disease activity also declined (Fig. 1f). Moreover, ESR scores declined from 120 mm/h to 78 mm/h in patient 1, from 120 mm/h to 34 mm/h in patient 2 and from 60 mm/h to 16 mm/h in patient 3, and C-reactive protein (CRP) also showed a marked reduction (Fig. 1g). DAS28 scores based on CRP decreased from 4.67 to 2.26 in patient 1, 4.04 to 2.67 in patient 2 and 4.61 to 1.56 in patient 3 at 24 weeks post-CAR T-cell infusion (Fig. 1h). Clinical disease activity index (CDAI) decreased from 23 to 1.5 points in patient 1, 18 to 2 points in patient 2 and 28 to 2 points in patient 3 (Fig. 1i). Simplified disease activity index (SDAI) decreased from 32.4 to 3.7 points in patient 1, 24.1 to 6.3 points in patient 2 and 29.4 to 2.1 points in patient 3 (Fig. 1i).

Consistent with clinical improvement, RA-associated autoantibodies significantly decreased over a 6-month period. Rheumatoid factor (RF) disappeared in all three patients. Robust decrease of anti-cyclic citrullinated peptide (CCP) antibodies was observed, from 399 RU/mL to 1 RU/mL in patient 1, from 865 RU/mL to 1 RU/mL in patient 2 and from 1604 RU/mL to 45 RU/mL in patient 3 (Fig. 1j). Follow-up Power Doppler ultrasound (PDUS) of the knee joints showed less effusion and Power Doppler (PD)-positive synovitis after the treatment (Fig. 1k). Moreover, improvement of synovitis was further confirmed by musculoskeletal Magnetic Resonance Imaging (MRI) scans, which showed regression of inflammation as evidenced by a decrease in the extent of osteitis, synovitis and tendinitis in the hands (Fig. 1l). Concerning long-term effects, we observed that all three patients showed B cell reconstitution at day 60, 90, and 60, respectively (Fig. 1d). Despite the reappearance of B cells, no relapse of RA was observed in the long-term follow-up of the patient 1 and 2 (9 months after CAR-T cell administration) (Supplementary information, Fig. S3a–c).

Immunoglobulin (Ig) levels consistently decreased. IgA levels dropped from 3.06, 5.48, and 3.12 g/L at baseline to 0.58, 0.82 and 0.80 g/L at the 24-week follow-up in patient 1, patient 2, and patient 3, respectively. IgG levels decreased from 10.40, 15.68, and 9.72 g/L to 8.74, 8.84, and 6.72 g/L, and IgM levels from 2.42, 4.55, and 1.39 g/L to 0.92, 0.91, and 0.50 g/L (Fig. 1m). As for cytokine profiles, patient 1 and 2 showed mild increase of IL-6 level, which might be explained by CAR T-cell expansion (Supplementary information, Fig. S4). Interestingly, we found that TNFα in patient 2 was normalized to baseline levels after CAR T-cell administration (Supplementary information, Fig. S4).

TNFα and IL-6 are crucial for triggering synovial inflammation and joint damage by orchestrating T helper cell differentiation, B cell proliferation and enhancement of osteoclastogenesis.^5,6^ Recently, Biesemann et al. found that inhibiting TNFα and IL-6 in combination could improve disease severity in a murine arthritis model over monotherapies.^7^ In our study, we provide an entirely new treatment approach whereby TNF inhibition, IL-6 inhibition and B-cell depletion are “packed” in one therapeutic cellular “machine”. This concept targets cytokine inhibition to sites of B-cell infiltrates in RA patients and thereby not only kills B-cells but provides local inhibition of inflammation. Such concept has not been introduced into human medicine so far. Besides, our fourth-generation CAR approach could not only foster the efficacy of the CAR T-cell in RA treatment by secreting anti-TNFα and anti-IL-6 but also abrogate CRS, which is frequently observed after CAR T-cell therapy due to activation of CAR T-cells.^8^ Indeed, we previously showed that aIL-6 scFv could successfully self-neutralize IL-6 release and thus minimize IL-6-associated cytokine toxicity.^9^ In conformity, in our study, we did not even observe mild forms of CRS.

B cells play an important role in RA and inhibition of B cells by anti-CD20 monoclonal antibody rituximab has proved to be efficacious in RA treatment.^10^ The three RA patients reported in this article have not been pre-exposed to rituximab, so one cannot conclude that B-cell targeting with rituximab would have not been effective in these patients. In practice, one would presumably pre-expose treatment-resistant RA patients with rituximab before a B-cell-targeted CAR treatment approach is used. However, only 30% of patients achieved a 50% improvement in the American College of Rheumatology response criteria after 6 months therapy of rituximab.^11^ In fact, CD19-targeting bispecific T cell engagers such as blinatumomab had shown to work even if rituximab and other B-cell-depleting antibodies have failed.^12^ This observation is likely based on the incomplete depletion of B cells in the tissues such as synovium, lymph nodes and the bone marrow by rituximab.^13,14^ Furthermore, in contrast to CD19, the CD20 antigen is not expressed on early B-cells and antibody-producing plasmablasts and plasma cells. The fast and robust decrease in autoantibody levels observed in the RA patients exposed to CAR T-cells supports a potential advantage of targeting CD19.

In summary, our study shows robust expansion of CAR T-cells in vivo, complete elimination of CD19 B-cells, clearance of RA-related autoantibodies, and high-level sustained clinical responses in RA patients, supporting a very recent observation in a patient with RA and coexisting myasthenia gravis.^15^ However, the patient reported by Haghikia et al.^15^ was not a typical RA patient refractory to multiple RA therapeutics. Therefore, our data show the feasibility of a fourth-generation CAR T-cell treatment approach in treatment-resistant RA with amelioration of inflammation and reduction of autoantibodies. This is the first time that a fourth-generation CAR has been used for the treatment of autoimmune disease and the rationale for combining cytokine inhibition and B-cell depletion in one approach bears huge opportunities to better control diseases like RA in the future.

While efficacy and safety look good in these patients showing the principal feasibility of this approach, it is clear that a general statement of the impact of this approach in the treatment of RA is too early since there are certain limitations in this study. First, only three RA patients were treated by this fourth-generation CAR and more patients are needed to validate its effect. Second, long-term observation is needed to determine the sustained efficacy and safety of this treatment. Nonetheless, these data show that RA is susceptible to CD19-CAR T-cell treatment and RA-specific autoimmunity can be eliminated by this approach. Therefore, it opens doors for new ways to comprehensively alleviate the pathology in a complex disease like RA.

## Supplementary information

Supplementary Information
