# Supplementary material for: Fourth-generation chimeric antigen receptor T-cell therapy is tolerable and efficacious in treatment-resistant rheumatoid arthritis
Source: Cell Res. 2025 Jan 9;35(3):220–3. doi: 10.1038/s41422-024-01068-2 (PMC11909189; doi:10.1038/s41422-024-01068-2)
Supplement: Supplementary file 1 — Supplementary Information [file 41422_2024_1068_MOESM1_ESM.pdf]

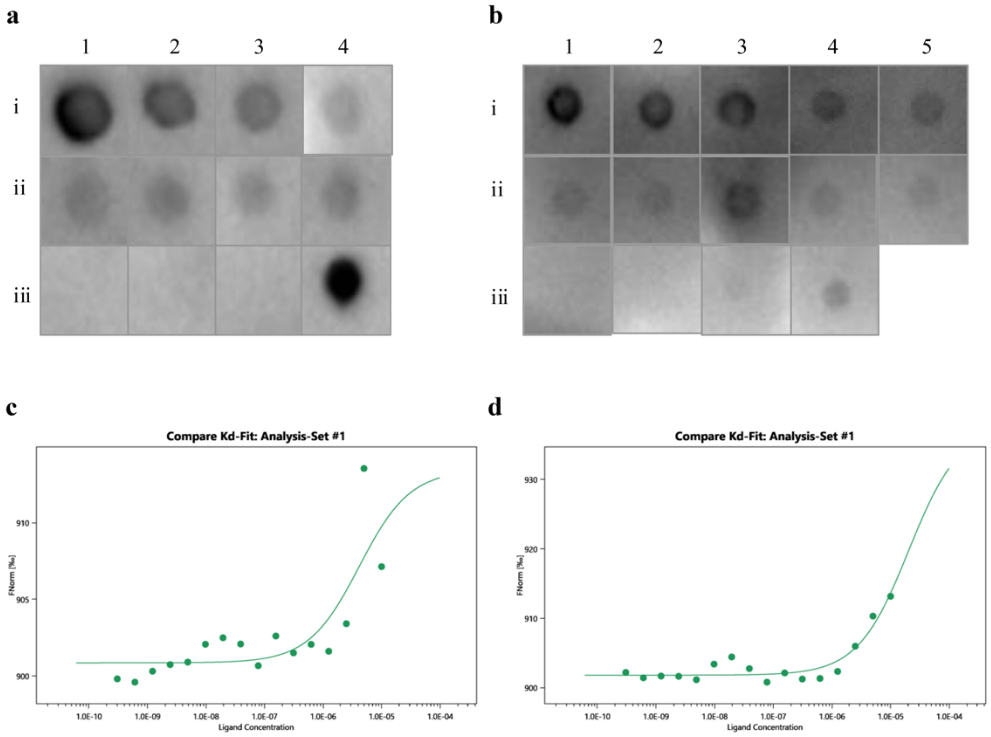

Fig. S1: anti-TNF (aTNFα) and anti-IL-6 (aIL-6) protein expression levels.

(a) Dot-blot analysis of aTNFα protein expression levels by the detection of aTNFα with a series of standard protein concentrations, labeled as i-1 to i-4 and ii-1 to ii-4, corresponding to 20 ng, 10 ng, 5 ng, 2.5 ng, 1.25 ng, 625 pg, 312.5 pg, and 156.25 pg, respectively. (b) Dot-blot analysis of aIL-6 protein expression levels by the detection of aIL-6 with a different set of standard concentrations, labeled similarly as i-1 to i-5 and ii-1 to ii-5, corresponding to 200 ng, 100 ng, 50 ng, 25 ng, 12.5 ng, 2 ng, 1 ng, 500 pg, 250 pg, and 125 pg, respectively. In (a) and (b), iii-1, iii-2, and iii-3 denote the negative controls consisting of PBS, 1640 medium, and T cell culture medium from Miltenyi Biotec, respectively. iii-4 in both panels represents the sample derived from 293F cells, serving as an experimental sample. The dot intensity is indicative of the relative protein concentration, providing a semi-quantitative measure of aTNFα and aIL-6 expression. (c-d), characterization of anti-cytokine binding in supernatant using the Molecular Surface Ligand (MSL) assay. The interaction between TNFα and aTNFα (c) in supernatant and that of IL-6 with aIL-6 (d), as indicated by the MSL binding curve.

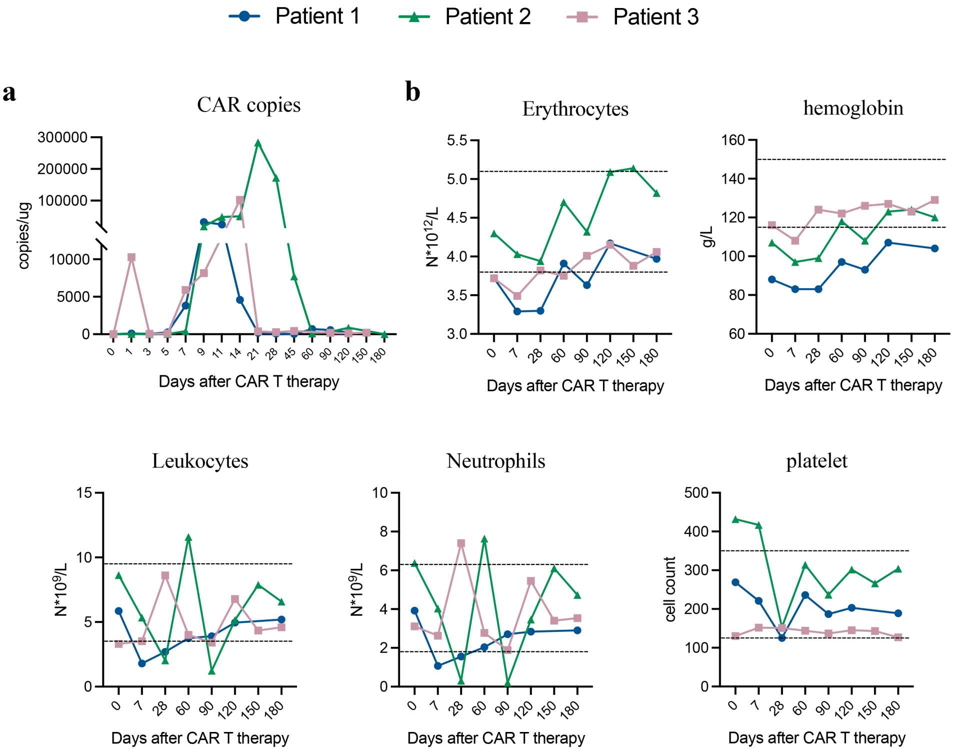

Fig. S2: Safety of CD19/aIL-6/aTNFα CAR T-cells in RA.

(a) Quantity of CAR copies in the patients' peripheral blood. (b) Numbers of blood cells lineages in the patients' peripheral blood.

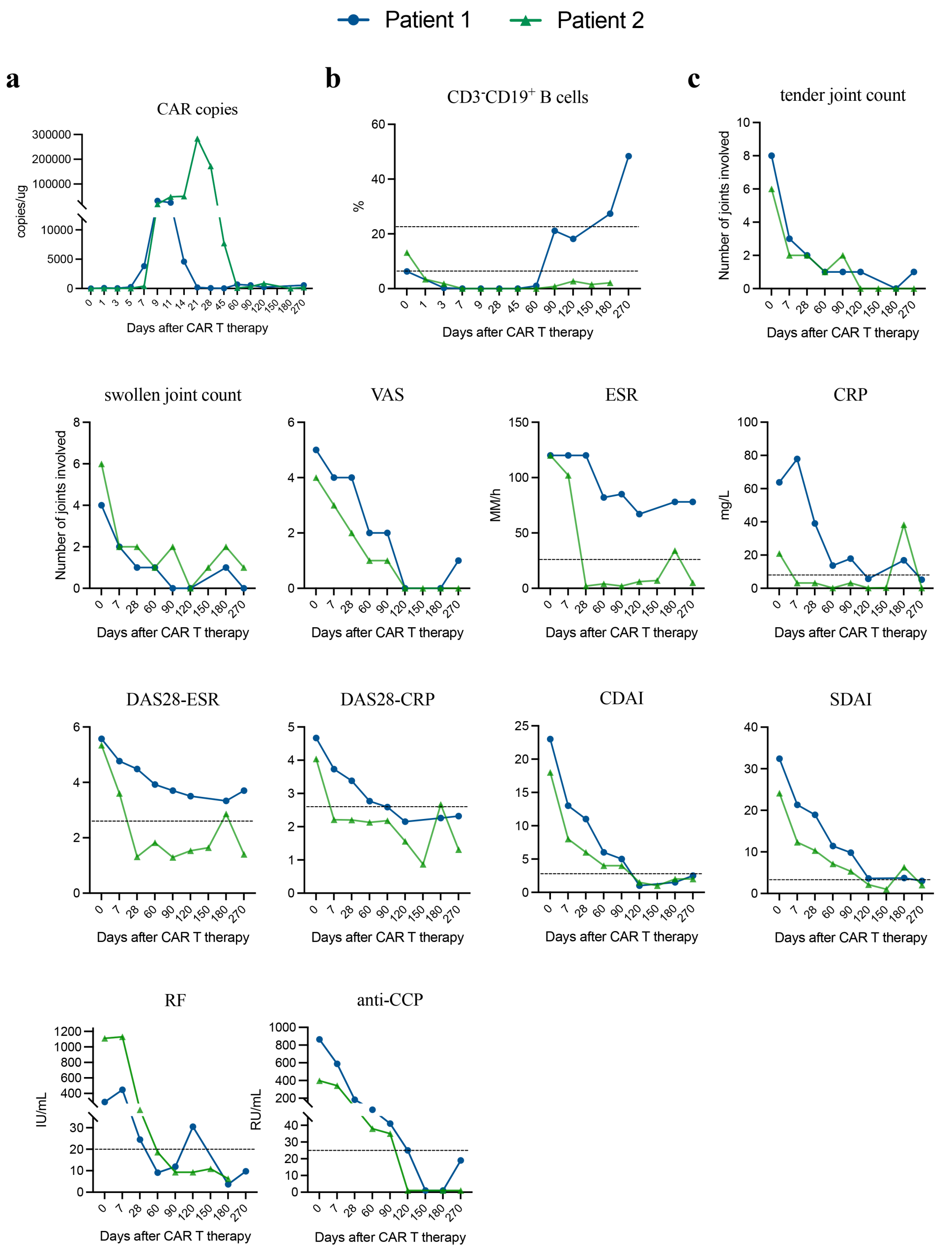

Fig. S3: Long-term efficacy of CD19/aIL-6/aTNFα CAR T-cells in RA.

(a-b) Numbers of CAR copies and circulating CD19$^+$ B cells in the patients' peripheral blood during 9-month follow-up. (c) Effects of CAR T-cell therapy on tender, swollen joint counts (TJC, SJC), visual analogue scale (VAS), C-reactive protein (CRP) levels, erythrocyte sedimentation rates (ESR), disease activity scores-28 (DAS28) based on CRP and ESR, clinical disease activity index (CDAI), simplified disease activity index (SDAI), serum levels of rheumatoid factor (RF) and antibodies against cyclic citrullinated peptide (CCP) at baseline and during 9-month follow-up.

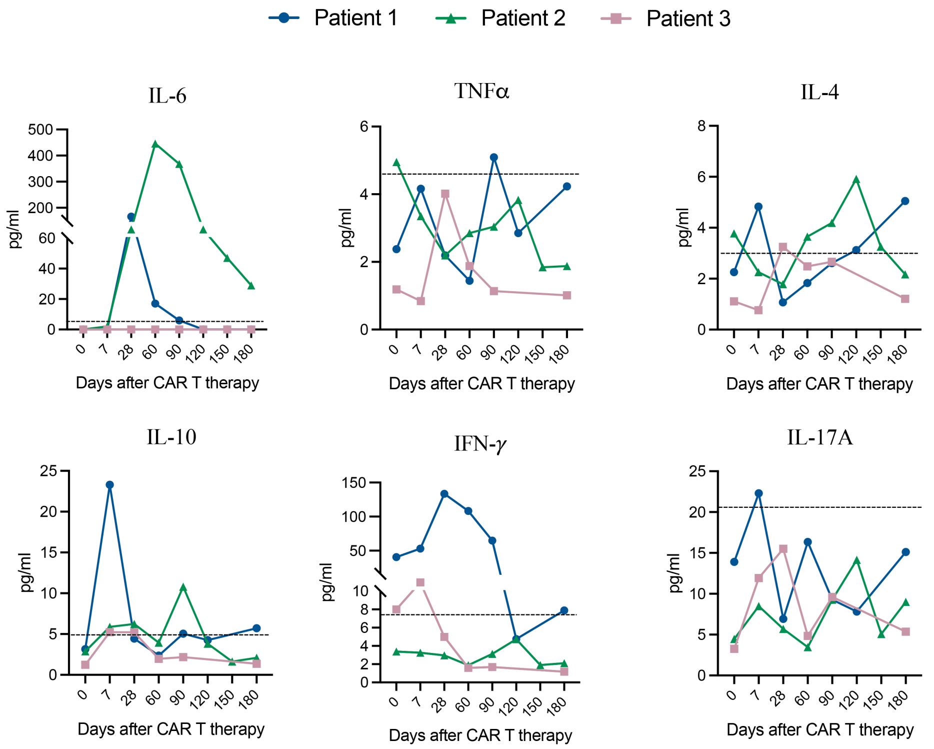

Fig. S4: Cytokine profiles in RA patients after CD19/aIL-6/aTNFα CAR T-cells infusion.

IL-6, TNFα, IL-4, IL-10, interferon-γ and IL-17A level during 6-month follow-up.

Table S1: Patient characteristics at baseline.

	Patient 1	Patient 2	Patient 3
**Demographics**			
Age	49	52	56
Gender	Female	Female	Female
**Disease status**			
Disease duration (years)	20	7	3
DAS28-ESR	5.57	5.34	5.90
ESR (mm/h)	120	120	60
CRP (mg/L)	63.77	20.93	3.2
RF (IU/ml)	290	1110.5	263
anti-CCP (RU/ml)	399	865	1604
**Medications**			
Methotrexate	+	+	+
Leflunomide	+	-	+
Hydroxychloroquine	+	-	+
Iguratimod	-	+	+
Glucocorticoid	+	+	+
Etanercept	+	+	-
Recombinant Human Tumor Necrosis Factor Receptor II:IgG Fc Fusion Protein	-	+	-
Adalimumab	+	-	-
Tofacitinib	+	-	-
Baricitinib	-	+	+
Abatacept	-	-	+

Table S2. Adverse events in patients.

	Patient 1	Patient 2	Patient 3
**≤Grade 2***			
Hypocalcemia	Yes	Yes	No
GGT increased	Yes	Yes	Yes
Hypoalbuminemia	No	Yes	No
Hypophosphatemia	Yes	No	No
Sinus tachycardia	Yes	Yes	No
Hypertriglyceridemia	Yes	Yes	Yes
Hyperuricemia	No	Yes	No
Alkaline phosphatase increased	Yes	Yes	No
Aspartate aminotransferase increased	Yes	Yes	Yes
Blood lactate dehydrogenase increased	No	Yes	Yes
Alanine aminotransferase increased	Yes	Yes	Yes
Cholesterol high	Yes	No	Yes
Fever	No	No	No
Neutrophil count decreased	Yes	Yes	No
White blood cell decreased	Yes	Yes	Yes
Hyperglycemia	No	No	Yes
Lymphocyte count decreased	Yes	Yes	Yes
Anemia	Yes	Yes	Yes
Hypokalemia	No	Yes	No
Hypoglycemia	No	Yes	No
Platelet count decreased	No	No	Yes
COVID-19 infection	No	Yes	No
**≥Grade 3***			
Lymphocyte count decreased	Yes	Yes	Yes
White blood cell decreased	Yes	Yes	No
Neutrophil count decreased	Yes	Yes	No
Alanine aminotransferase increased	No	Yes	No
GGT increased	No	Yes	Yes
Hyponatremia	No	Yes	No
Anemia	Yes	No	No
Hypokalemia	No	Yes	No

* Evaluated by Common Terminology Criteria for Adverse Events (CTCAE) version 5.0

Methods

Patient selection and enrollment.

Patients with D2T RA were recruited at Department of Rheumatology and Immunology, the First Affiliated Hospital of University of Science and Technology of China between November 2023 and February 2024. Eligibility for patient inclusion was based on (1) age 18 years or older, (2) a diagnosis of RA according to the American College of Rheumatology–European League against Rheumatism (ACR–EULAR) 2010 criteria, (3) evidence for B-cell involvement in disease based on positivity of rheumatoid factor (RF) or anti-cyclic citrullinated peptide (CCP), (4) treatment resistance to at least two different targeted synthetic or biological disease-modifying antirheumatic drugs (DMARDs) and (5) no concomitant severe cardiovascular disease, active infection or immunodeficiency. Three patients with D2T RA were selected for CD19/anti-IL-6/anti-TNFα (aIL-6/aTNFα) chimeric antigen receptor (CAR) T-cell therapy by specialized rheumatologists. An interdisciplinary team, consisting of rheumatologists and cellular therapy specialists decided upon the eligibility for patient use of CD19/aIL-6/aTNFα CAR T-cells considering the patients′ disease severity, treatment resistance, progression and overall prognosis. The protocol was approved by the ethics committee of the First Affiliated Hospital of University of Science and Technology of China (2023KY-379). All participants gave written informed consent for all the procedures and the data sharing in compliance with the principles of the Declaration of Helsinki.

Plasmids and retroviral transduction

To construct CARs, we utilized a CD19-41BB-CD3ζ backbone, previously established in our lab. Two single-chain variable fragment (scFv) sequences, namely aIL-6 derived from Sirukumab and aTNFα derived from Adalimumab, were genetically fused to the CAR structure using T2A and P2A linkers, respectively. These coding sequences were cloned into the lentivector. Subsequently, the CAR-expressing vectors were utilized to generate lentiviral particles for transduction.

CD19/aIL-6/aTNFα CAR T-cells manufacturing and treatment

Peripheral blood was collected from the enrolled patients and processed with Ficoll (GE Healthcare) gradient centrifugation to isolate peripheral blood mononuclear cells (PBMC) at day 0. T cells were enriched and activated with anti-CD3&CD28 dynabeads (Thermo Fisher) for 24 hours and then transduced with 4th generation lentivector encoding anti-CD19 CAR and anti-IL-6/TNFα single chain variable fragment (scFv) (Fig. 1a, b). After 48 hours, the dynabeads were removed. CAR T-cells were further expanded and analyzed for CAR expression (Novocyte, Agilent). The CAR T-cells were also tested for sterility and in vitro killing of leukemia cells Nalm6 or K562 cells (ATCC) expressing GFP (Novocyte, Agilent). At day 10-14, CAR T-cells were collected and prepared.

RA patients were admitted to the hospital for receiving lymphodepleting chemotherapy with fludarabine (25 mg/m^2 [-5 to -3 days]) and cyclophosphamide (300 mg/m^2 [-5 to -3 days]), followed by single infusion of 1.0×10^6/kg CAR T-cells. Patients were monitored every day for signs of cytokine -release syndrome (CRS) during the first 14 days after administration of CAR T-cell treatment.

aTNFα and aIL-6 protein expression levels.

Following the infusion of CAR T-cells into patients, the levels of those anti-cytokines in the systemic circulation were exceedingly low, primarily due to their rapid clearance and short half-lives. This inherent difficulty in detecting these bioactive molecules at an optimal time point in the bloodstream necessitates alternative strategies for their assessment. To circumvent these in vivo detection limitations, we employed an in vitro approach to validate the expression of aTNFα and aIL-6 by CAR T-cells. Utilizing Dot blot assays in the supernatants from in vitro cultured CAR T-cells, we successfully detected the presence of both aTNFα and aIL-6. The anti-cytokines secreted by CAR T-cells were measured in the supernatants from in vitro cultured CAR T-cells, thereby confirming their production in an environment that closely mimics the physiological conditions.

Furthermore, to evaluate the functional capacity of the secreted anti-cytokines, Molecular Surface Ligand (MSL) binding assays were conducted. The MSL method is a robust technique for assessing the binding affinity and specificity of ligands to their respective receptors. Our results demonstrated that both aTNFα and aIL-6 exhibited binding capabilities to their target cytokines, TNFα and IL-6, respectively. The binding curves generated from the MSL assays provided quantitative insights into the interaction dynamics, confirming the biological relevance of the CAR T-cell-derived anti-cytokines.

Clinical Assessments and Follow-ups.

CAR vector copy numbers were assessed by quantitative PCR using DNA purified from patient blood sample by gDNA isolation kit (TIANGEN) and analyzed with SYBR Green qPCR kit (Takara) according to manufacturer's protocol on Lightcycler (Roche). RA disease activity was assessed by Disease Activity Score-28 (DAS28) score, Simplified Disease Activity Index (SDAI) and Clinical Disease Activity Index (CDAI). Patients were assessed monthly, including CAR T-cell and blood cell counts, blood biochemistry, and serum levels of autoantibodies, immunoglobulin and cytokines. Power Doppler (PD) ultrasound (US) of knee joints and magnetic resonance imaging (MRI) assessment of hands were performed at indicated time points.
